# Intra-Articular Injection of Human Synovial Membrane-Derived Mesenchymal Stem Cells in Murine Collagen-Induced Arthritis: Assessment of Immunomodulatory Capacity In Vivo

**DOI:** 10.1155/2017/9198328

**Published:** 2017-06-21

**Authors:** Minglu Yan, Xin Liu, Qiujie Dang, He Huang, Fan Yang, Yang Li

**Affiliations:** Department of Rheumatology and Immunology, The Second Affiliated Hospital of Harbin Medical University, Harbin 150000, China

## Abstract

The aim of this study was to evaluate the efficacy of human synovial membrane-derived MSCs (SM-MSCs) in murine collagen-induced arthritis (CIA). Male mice (age 7–9 weeks) were injected intra-articularly with SM-MSCs obtained from patients with osteoarthritis, on days 28, 32, and 38 after bovine type II collagen immunization. The efficacy of SM-MSCs in CIA was evaluated clinically and histologically. Cytokine profile analyses were performed by real-time polymerase chain reaction and multiplex analyses. Splenic helper T (Th) cell and regulatory B cell subsets were analyzed by flow cytometry. Intra-articular SM-MSC injection ameliorated the clinical and histological severity of arthritis. Decrease in tumor necrosis factor-*α*, interferon-*γ*, and interleukin- (IL-) 17A and increase in IL-10 production were observed after SM-MSC treatment. Flow cytometry showed that Th1 and Th17 cells decreased, whereas Th2, regulatory T (Treg), and PD-1^+^CXCR5^+^FoxP3^+^ follicular Treg cells increased in the spleens of SM-MSC-treated mice. Regulatory B cell analysis showed that CD21^hi^CD23^hi^ transitional 2 cells, CD23^low^CD21^hi^ marginal zone cells, and CD19^+^CD5^+^CD1d^+^IL-10^+^ regulatory B cells increased following SM-MSC treatment. Our results demonstrated that SM-MSCs injected in inflamed joints in CIA had a therapeutic effect and could prevent arthritis development and suppress immune responses via immunoregulatory cell expansion.

## 1. Introduction

Rheumatoid arthritis (RA) is a systemic autoimmune disorder characterized by persistent inflammation, extensive synovial hyperplasia, and, ultimately, cartilage and bone destruction [1]. Loss of self-tolerance leads to imbalance of effector and regulatory cells, which plays a crucial role in the onset and pathogenesis of RA [2, 3]. In particular, interferon- (IFN-) *γ*-helper T (Th) 1 and interleukin- (IL-) 17-Th17 cells are thought to be the etiologic populations, whereas regulatory T (Treg) cells and B cells, with an IL-10-secreting profile, are capable of recovering self-tolerance and preventing autoimmune diseases [4–6]. Hence, the recovery of immune tolerance by expansion of regulatory cells may be a rational approach for RA treatment.

MSCs are adult multipotent cells that are present in the bone marrow (BM), adipose tissue, synovial membrane, synovial fluid, and perinatal tissues. These cells have been characterized with respect to colony-forming unit fibroblast (CFU-F), surface marker expression, and in vitro multidifferentiation potential, according to the International Society for Cellular Therapy (ISCT) criteria [7]. During the last several decades, MSCs have been largely investigated for their potent immunomodulatory and anti-inflammatory capacities, emerging as a promising therapy for autoimmune diseases such as RA [8–10].

Although an accumulating body of work clearly demonstrates that MSCs harvested from BM and placental cultures possess potent immunomodulatory effects in vitro and in vivo [11–13], relative little is known about the role of synovial membrane-derived MSCs (SM-MSCs) in the immune system. SM-MSCs were first identified in 2001; it was reported that the synovial membrane from the knee joints of human donors could give rise to a fibroblast-like cell population possessing great expansion potential, typical antigen expression, and multidifferentiation capability [14]. Recently, several studies demonstrated that SM-MSCs from patients with osteoarthritis (OA) could suppress T cell proliferation and maintain the Treg population in vitro when cocultured with allogeneic lymphocytes [15, 16], suggesting that SM-MSCs can also be employed to develop a distinct immunomodulatory approach. However, the in vivo regulatory role of SM-MSCs in RA is yet unclear.

In this study, we employed a murine collagen-induced arthritis (CIA) model to evaluate the therapeutic effect of SM-MSCs following repeated intra-articular injection. To our knowledge, this study is the first to show that SM-MSCs can exert immunomodulatory effects in CIA via expansion of FoxP3^+^ Treg cells and CD21^hi^CD23^hi^ transitional 2 (T2), CD23^low^CD21^hi^ marginal zone (MZ), and IL-10-competent regulatory B cells. Our data indicate that SM-MSC administration may provide a promising approach for RA treatment.

## 2. Materials and Methods

### 2.1. Isolation and Expansion of MSCs from Human Synovial Membranes

MSCs were isolated from human synovial membranes as previously described [17]. Synovial membranes were obtained aseptically from the knee joints of human donors (age 64 ± 8 years, 21 females and 18 males) at the time of surgical knee replacement for degenerative OA at the Second Affiliated Hospital of Harbin Medical University, with the donors' understanding and informed consent. Exclusion criteria for these donors were rheumatic diseases, infections at the time of this study, and a history of malignancy.

Synovial membranes were rinsed twice in Hank's balanced salt solution (HBSS; Hyclone) supplemented with antibiotic-antimycotic solution (100 U/mL penicillin, 100 *μ*g/mL streptomycin, and 0.25 *μ*g/mL amphotericin B, Life Technologies), finely minced, and digested with 0.2% type I collagenase (Life Technologies) in Dulbecco's modified Eagle's medium-low glucose (DMEM-LG; Hyclone) containing 10% fetal bovine serum (FBS; Excell Bio) and 1% penicillin/streptomycin (P/S; Invitrogen). Following 8 h incubation at 37°C, undigested tissues were removed using a 70 *μ*m nylon sieve, and cells were collected, washed twice, resuspended in DMEM-LG supplemented with 10% FBS and 1% P/S solution (referred to as growth medium), and plated in a T25 culture flask for expansion at 37°C in a humidified 5% CO_2_ atmosphere for 3-4 days. Nonadherent cells were removed, and the growth medium was refreshed every 3 days until confluence was achieved. The MSC monolayer was detached using trypsin-ethylenediaminetetraacetic acid (EDTA) (0.25% trypsin, 0.53 mM EDTA; Invitrogen) and subsequently passaged twice before use.

For the CFU-F assay, cells were seeded at a density of 10^4^ cells/well in 6-well plates and cultured in growth medium for 10 days. The cells were subsequently fixed and stained with 0.5% crystal violet in 4% paraformaldehyde for 5 min. All visible colonies were counted.

### 2.2. Identification of SM-MSCs

The immunophenotype of SM-MSCs was identified by flow cytometry analysis (FACS Canto II, BD Biosciences) by using the following fluorescent antibodies: phycoerythrin- (PE-) conjugated mouse antihuman CD34, CD45, and CD90 antibodies; fluorescein isothiocyanate- (FITC-) conjugated CD73 antibodies; and allophycocyanin- (APC-) conjugated CD105 antibodies. As an isotype control, the appropriate mouse immunoglobulin (Ig) G_1_ was substituted for the primary antibody. All the antibodies were purchased from BD Pharmingen (San Diego, CA, USA).

SM-MSCs were next tested for their capacity to differentiate toward the adipogenic and osteogenic lineages. For adipogenic induction, 2.5 × 10^5^ MSCs were plated in a 6-well plate and treated with hMSC Adipogenic Differentiation BulletKit™ Medium (Lonza) and maintained for 14 days before being subjected to Oil Red O staining (Sigma-Aldrich). For osteogenic induction, MSCs were digested and seeded in a 6-well plate at a density of 10^5^ cells/well and then maintained in hMSC Osteogenic Differentiation BulletKit Medium (Lonza) for 21 days before being subjected to Alizarin Red S staining (Sigma-Aldrich).

### 2.3. Induction of CIA and SM-MSC Treatment

CIA was induced in male DBA/1J mice (age 7–9 weeks) purchased from Shanghai Laboratory Animal Center (SLAC, Shanghai, China). The mice (*n* = 20) were maintained under standard conditions in our university's central animal laboratory and randomly placed in cages. Bovine type II collagen (CII; Chondrex) was dissolved in 50 mM acetic acid and emulsified with an equal volume of Freund's complete adjuvant (Sigma-Aldrich). The mice were immunized at the base of the tail with 100 *μ*L emulsion containing 200 *μ*g CII. After 21 days, the mice were administered a booster dose of 100 *μ*g CII (2 mg/mL) emulsified with Freund's incomplete adjuvant (Chondrex) via intradermal injection into the tail. On days 28, 32, and 38 after the first immunization, the mice were anesthetized and 10^6^ MSCs in 7 *μ*L PBS were injected intra-articularly into the right knee for the SM-MSC treatment group (*n* = 8); the control mice received 7 *μ*L PBS intra-articularly (*n* = 8).

### 2.4. Clinical and Histological Assessment of Arthritis

Clinical arthritis and severity scores of each individual mouse (*n* = 8 per group) were evaluated every 2 days by using the mean arthritis severity index, with scores on a scale of 0–4, as previously reported [18]. The mean thickness of the hind paw was measured with vernier calipers. The mice were anesthetized and euthanized on day 70 after CII immunization, and the ankle joints (right) were harvested for histological assessment. The joints were fixed in 4% paraformaldehyde, decalcified in 10% EDTA for 48 h, and embedded in paraffin. Tissues were sectioned at 7 *μ*m thickness and stained with hematoxylin and eosin (H&E). All stained joint sections were observed in a blinded manner with light microscopy.

### 2.5. Quantitative Polymerase Chain Reaction (PCR)

The synovia from the right knee joints of the mice was harvested at the end of the experiment. Total RNA was extracted using TRIzol reagent (Invitrogen) and reverse transcribed to cDNA with a Primescript RT Kit (Takara) according to the manufacturer's instructions. SYBR Green-based real-time PCR was performed using the 7500 Fast Real-Time PCR System (Applied Biosystems, USA) to quantify the mRNA levels of TNF-*α*, IFN-*γ*, IL-17A, IL-10, IL-4, and transforming growth factor- (TGF-) *β*. Relative changes in gene expression were calculated using the comparative C_T_ method. The mRNA levels of the target genes were normalized to those of the *β*-actin gene. Sequences of primers used in this study were obtained from primer bank of Harvard University and were listed in Table 1.

### 2.6. Multiplex Analysis

Peripheral blood samples were collected from the angular vein of mice on day 70 after etherization, and serum was obtained following a standard protocol. The cytokine (TNF-*α*, IFN-*γ*, IL-17A, IL-10, and IL-4) concentrations in serum were determined with the Multiplex Cytokine Bead Array System (Merck, Germany) according to the manufacturer's instructions.

### 2.7. Flow Cytometry

Splenocytes were freshly prepared on day 70, and single-cell suspensions were stained with the following antibodies for cell surface analysis: anti-CD4-FITC, anti-PD-1-peridinin chlorophyll (PerCP), anti-CXCR5-APC, anti-B220-APC, anti-CD21-FITC, anti-CD23-PE, anti-CD19-PerCP, anti-CD5-FITC, and anti-CD1d-APC antibodies. For transcription factor staining, the cells were fixed and permeabilized using a commercial FoxP3 staining kit (eBioscience, USA) according to the manufacturer's protocols. For intracellular cytokine staining, isolated splenocytes were stimulated with phorbol myristic acetate (PMA; 50 ng/mL)/ionomycin (1 *μ*g/mL) for 4 h in the presence of brefeldin (3 *μ*g/mL) and monomycin (1.4 *μ*g/mL). The following antibodies were used: anti-IFN-*γ*-PE, anti-IL-4-PE, anti-IL-17-PE, and anti-IL-10-PE antibodies. Appropriate isotype-matched control antibodies were used to determine nonspecific staining. All the antibodies were bought from BD Biosciences. Data were acquired with a FACS Canto II flow cytometer (BD Biosciences, USA) and analyzed using the FlowJo software.

### 2.8. Statistical Analysis

Data were presented as mean ± standard deviation (SD). Comparisons of parametric data between the two groups were analyzed with Student's *t-*test. Statistical analysis was performed with GraphPad Prism (version 6 for Mac; GraphPad Software). *p* values less than 0.05 were considered significant.

### 2.9. Ethics Statement

The use of human materials in this study was approved by the Medical Ethical Committee of Harbin Medical University. All mouse experiments were conducted with the permission of the local ethics committee on animal research and were in compliance with the national guidelines for laboratory animal use.

## 3. Results

### 3.1. Characterization of Human SM-MSCs

**Figure 1:**
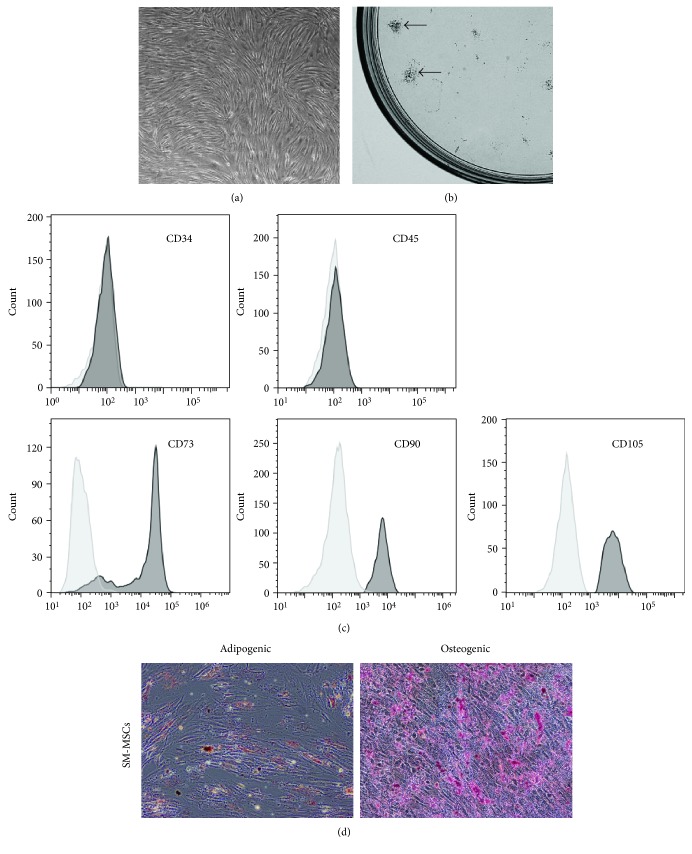
Characterization of MSCs isolated from human synovial membranes. (a) SM-MSCs from passage 3 exhibited a typical spindle-shaped morphology under an inverted microscope. (b) Representative colony-forming unit analysis. (c) Phenotypic analysis of SM-MSCs by flow cytometry. Histograms showed levels of surface antigen expression and their corresponding isotype control. (d) Multilineage differentiation potential of SM-MSCs. Samples were stained with Oil Red O, indicating differentiated adipocytes, and with Alizarin Red S staining, indicating mature osteoblasts. Original magnification, ×100. SM-MSCs, synovial membrane-derived MSCs.

The SM-MSCs displayed a spindle-like, fibroblast morphology (Figure 1(a)), which is a typical characteristic of MSCs. Since the CFU-F assay is considered to provide the closest estimate of MSC levels [19], we evaluated the colony-forming efficacy of cells isolated from the human synovial membranes. The cells developed large colonies as the culture continued for 10 days (Figure 1(b)), suggesting the high yield and expansion potential of the isolated MSCs. Flow cytometry analysis revealed that SM-MSCs were negative for the hematopoietic lineage markers CD34 and CD45, whereas they were positive for CD73, CD90, and CD105 (Figure 1(c)). SM-MSCs were able to differentiate toward mature adipocytes and osteocytes revealed by Oil Red O staining and Alizarin Red S staining (Figure 1(d)).

### 3.2. Administration of SM-MSC Ameliorated Collagen-Induced Arthritis

**Figure 2:**
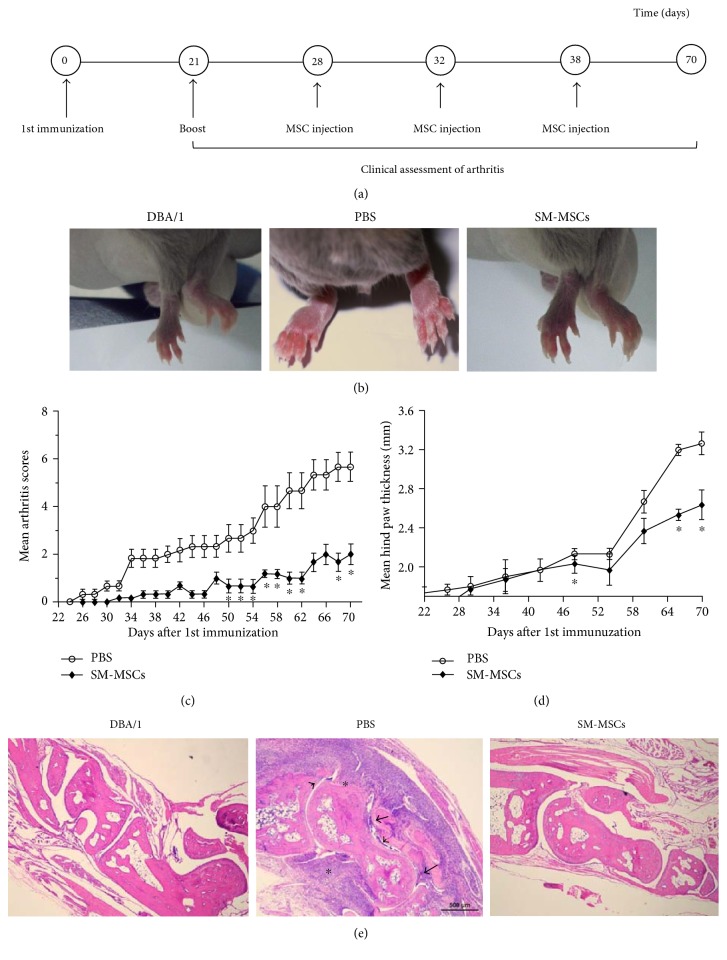
Decrease in severity of CIA following SM-MSC treatment. (a) Experimental design. DBA/1 mice were subcutaneously immunized with 200 *μ*g CII in Freund's complete adjuvant on day 0 and administered a booster dose via intradermal injection of 100 *μ*g CII into the tail on day 21. The CII-immunized mice were treated three times with SM-MSCs (10^6^) in 7 *μ*L PBS or PBS alone intra-articularly when arthritis had been established (*n* = 8 per group). The arthritis index score and hind paw thickness of mice in each group were recorded following booster immunization. (b) Representative photos of the paws in normal mice, PBS-treated mice, and SM-MSC-treated mice. (c) Arthritis severity was scored every 2 days, and (d) hind paw thickness was measured every 6 days. (e) Histological sections of the ankle joints (right) were stained with H&E (magnification, ×40). The asterisks denote the presence of inflammatory infiltrates, the arrows indicate synovial hyperplasia, and the arrowheads show the formation of pannus layer. Data have been presented as mean±SD values. ^∗^*p* < 0.05, compared with corresponding time point.

We injected the prepared cells intra-articularly into the right knees of the mice on days 28, 32, and 38 after the first immunization (Figure 2(a)). Repeated intra-articular injection of 10^6^ SM-MSCs in the right knee efficiently attenuated the arthritis symptoms (Figure 2(b)) and decreased the mean arthritis scores (*p* < 0.05) (Figure 2(c)). The hind paw thicknesses of SM-MSC-injected mice were significantly lower than those of control mice (*p* < 0.05) (Figure 2(d)). Histologically CIA treated with PBS was characterized by the accumulation of inflammatory infiltrates in the synovial tissue, synovial hyperplasia of the synovial lining layer, followed by the formation of pannus and joint damage. Histological analysis of the ankle joints in SM-MSC-treated mice revealed a rather normal joint architecture, exhibiting markedly decreased cellular infiltration, without or limited synovitis and pannus formation (Figure 2(e)). Thus, repeated intra-articular injection of SM-MSCs exerted a profound therapeutic effect in CIA.

### 3.3. Reprogramming Cytokine Profiles following SM-MSC Treatment

**Figure 3:**
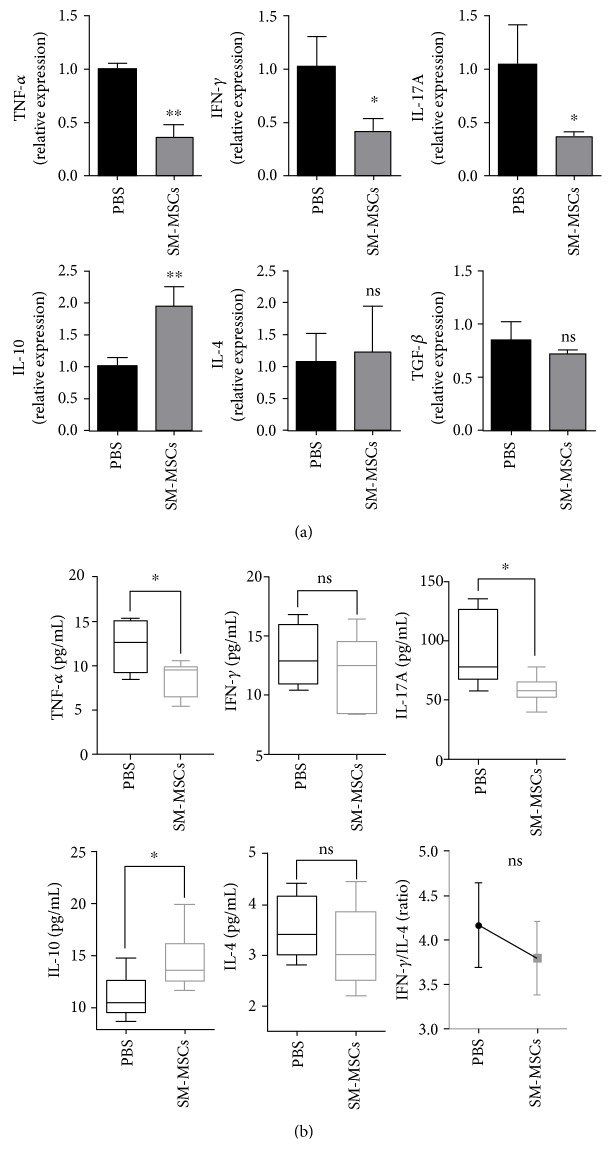
Effect of local SM-MSC administration on cytokine profiles in CIA mice. (a) Synovia of the right knee joints was harvested from five mice of each group at the end of the experiment, and quantitative PCR was performed to measure mRNA levels of several cytokines in resident synoviocytes. The mRNA levels of the target genes were normalized to those of the *β*-actin gene. (b) Peripheral blood from SM-MSC-treated mice and PBS-treated mice was obtained on day 70, and serum samples were tested for TNF-*α*, IFN-*γ*, IL-17A, IL-10, and IL-4 concentrations by using Milliplex analysis. The IFN-*γ*/IL-4 ratio was calculated. Data have been shown as mean±SD values. ^∗^*p* < 0.05, ^∗∗^*p* < 0.01; ns, no significance.

We analyzed the cytokine gene expression of resident synoviocytes in the absence or presence of allogeneic SM-MSCs. The synovial tissues (right knee joints) were harvested from the mice on day 70, and mRNA levels of TNF-*α*, IFN-*γ*, IL-17A, IL-10, IL-4, and TGF-*β* were quantified. Compared with the findings for PBS-treated controls, the synoviocytes in the presence of SM-MSCs showed decreased TNF-*α*, IFN-*γ*, and IL-17A expression (TNF-*α*: *p* = 0.0013, IFN-*γ*: *p* = 0.0268, and IL-17A: *p* = 0.0323), accompanied with an important increase in IL-10 transcripts (*p* = 0.0067). IL-4 and TGF-*β* expression did not significantly differ between the SM-MSC-treated and PBS-treated groups (IL-4: *p* = 0.7568, TGF-*β*: *p* = 0.3141) (Figure 3(a)).

Next, we analyzed the cytokine concentrations in serum. Multiplex analysis revealed that TNF-*α* and IL-17A concentrations in serum significantly decreased in SM-MSC-treated mice, whereas the titers of the anti-inflammatory mediator IL-10 significantly increased (TNF-*α*: *p* = 0.0264, IL-17A: *p* = 0.0424, and IL-10: *p* = 0.0438). No significant difference was observed in IFN-*γ* and IL-4 levels after SM-MSC treatment in CIA mice (IFN-*γ*: *p* = 0.4603, IL-4: *p* = 0.3976). However, the IFN-*γ*/IL-4 ratio was much lower in mice treated with SM-MSCs than in PBS controls (*p* = 0.1845) (Figure 3(b)). Collectively, these data demonstrated that local administration of SM-MSCs resulted in a systemic regulatory effect on cytokine profiles in CIA mice.

### 3.4. SM-MSC Treatment Corrected the Balance between Th1/Th17 and FoxP3-Expressing Treg Cells in the Spleen

**Figure 4:**
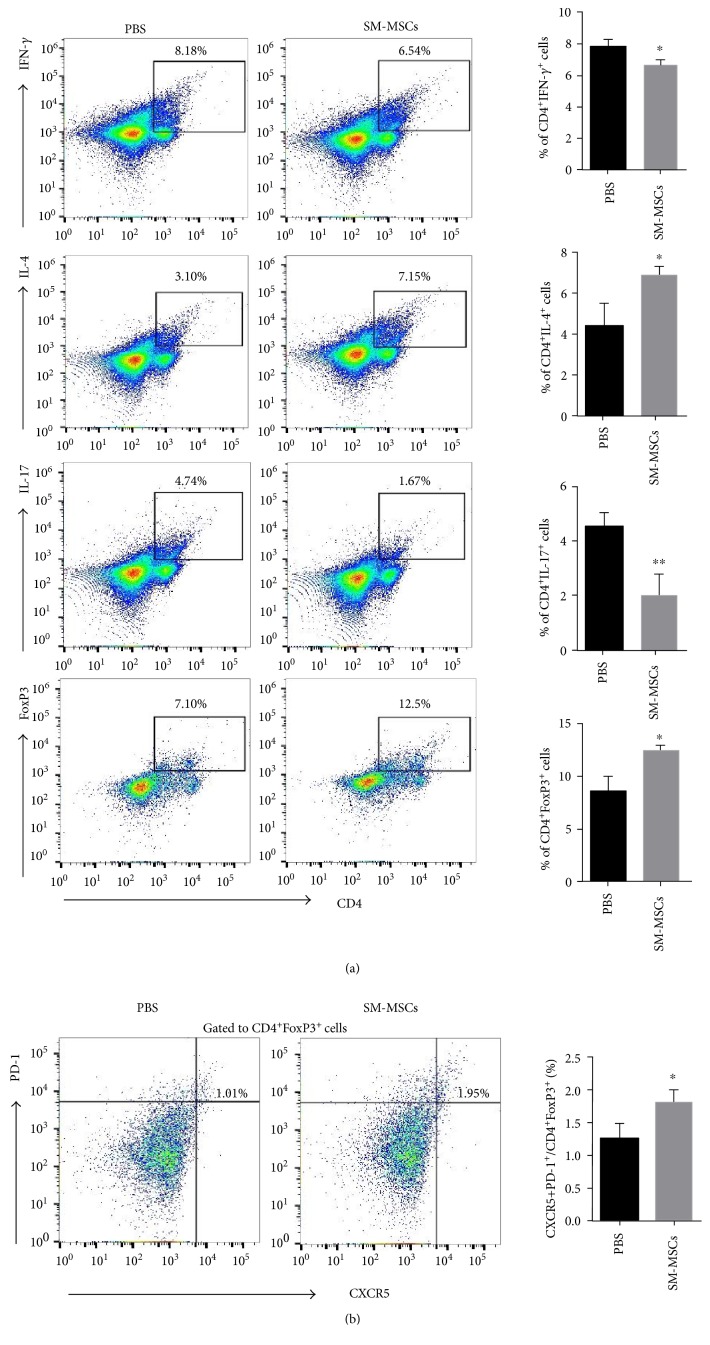
Effect of SM-MSC treatment on the frequency of Th cell subsets in mice with CIA. Splenocytes from the mice in each group (*n* = 5 per group) were prepared and stimulated with PMA/ionomycin for 4 h, following which they were analyzed by flow cytometry. (a) Left, frequencies of Th1 (CD4^+^IFN-*γ*^+^), Th2 (CD4^+^IL-4^+^), Th17 (CD4^+^IL-17^+^), and Treg (CD4^+^FoxP3^+^) cells in the spleens of mice treated or not treated with SM-MSCs. Right, corresponding bar graphs show quantification of cell percentages. (b) Left, Tfr cells were assessed for the expression of PD-1^+^CXCR5^+^ cells gated from CD4^+^FoxP3^+^ cells by flow cytometry. Right, quantification of the percentage of CD4^+^CXCR5^+^PD-1^+^FoxP3^+^ cells. Data have been presented as mean±SD values. ^∗^*p* < 0.05, ^∗∗^*p* < 0.01.

Dysregulated Th cell subsets have been proved to be major players in triggering the cytokine cascade responsible for tissue injury [20]. Hence, we next addressed whether repeated SM-MSC treatment for mice with CIA could correct the balance of Th cell subsets in the spleen. Splenocytes were isolated and stimulated with PMA/ionomycin before flow cytometry analysis. The frequency of Th1 cells, defined as IFN-*γ*-expressing CD4^+^ T cells, was significantly lower in SM-MSC-treated mice than in PBS-treated mice, whereas Th2 cells, defined as IL-4-expressing CD4^+^ T cells notably increased (Th1: *p* = 0.0190, Th2: *p* = 0.0247). Moreover, there were fewer IL-17-expressing CD4^+^ T cells and much more FoxP3^+^CD4^+^ Treg cells in SM-MSC-treated mice (Th17: *p* = 0.0087, Treg: *p* = 0.0121) (Figure 4(a)). Thus, SM-MSCs could help recover the Th1/Th2 and Th17/Treg cell balance in CIA.

The critical enhancement of FoxP3^+^ T cells by SM-MSCs prompted further analysis of the subpopulation of Treg cells. Follicular Treg (Tfr) cells, originating from natural Treg precursors, have been documented to have a suppressive effect upon T cell proliferation in vitro and germinal center (GC) B cell responses in vivo [21, 22]. In our study, a significant increase in CD4^+^CXCR5^+^PD-1^+^FoxP3^+^ Tfr cells was observed in SM-MSC-treated mice as compared to PBS controls (*p* = 0.0368) (Figure 4(b)). Together, these data indicated that increase in FoxP3-expressing Treg cells, accompanied by decrease in Th1 and Th17 responses, was involved in the mechanisms underlying arthritis remission under SM-MSC treatment.

### 3.5. SM-MSCs Favored the Development of Regulatory B Cells in the Spleen

**Figure 5:**
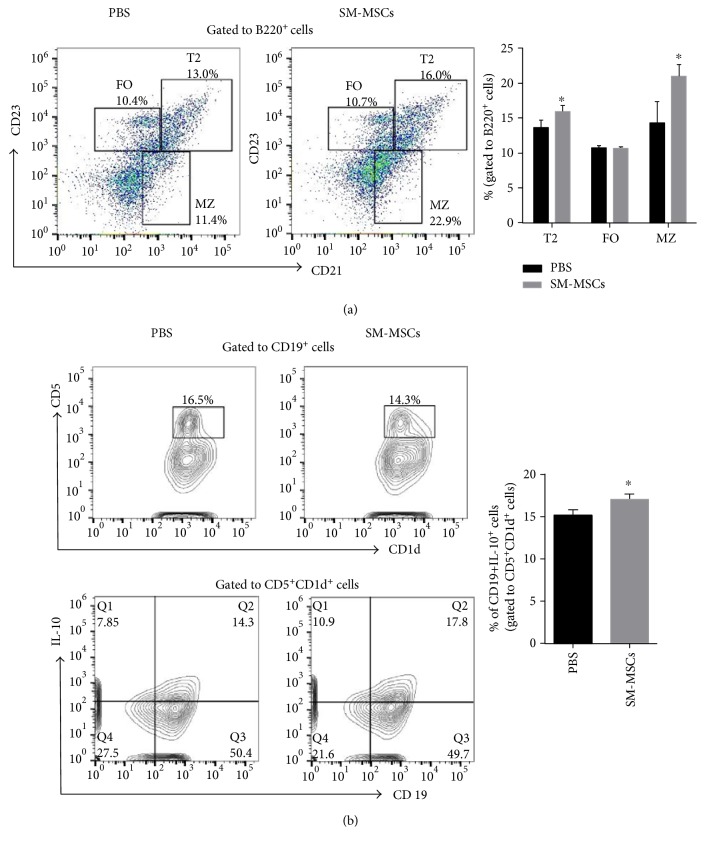
SM-MSCs favored the development of regulatory B cells in the spleen. (a) Surface expression of CD21 and CD23 on B220^+^ gated B cells derived from mice treated or not treated with SM-MSCs (*n* = 5 per group). The proportions of T2 (CD21^hi^CD23^hi^), FO (CD21^int^CD23^int^), and MZ (CD23^low^CD21^hi^) cells were analyzed by flow cytometry. (b) Splenocytes were stimulated with PMA/ionomycin for 4 h and analyzed for the frequency of B10 (CD19^+^CD5^+^CD1d^+^IL-10^+^) cells in mice. Data have been presented as mean±SD values. ^∗^*p* < 0.05.

Previous research showed that regulatory B cells play a key role in the maintenance of peripheral tolerance via Th1 and Th17 response inhibition and FoxP3^+^ Treg cell pool induction [23]. Furthermore, Tfr cells in the spleen require regulatory B cells for optimal expansion and differentiation [24]. To determine whether increase in Treg cells in SM-MSCs-treated mice was accompanied by increase in regulatory B cells, we analyzed the proportion of several B cell subsets that have previously been characterized in the case of mechanisms underlying immune tolerance [25]. B220^+^ cells were phenotypically analyzed for CD21 and CD23 expression. SM-MSC-treated mice had a considerably higher frequency of CD21^hi^CD23^hi^ T2 cells and CD23^low^CD21^hi^ MZ cells than PBS controls, while the proportion of CD21^int^CD23^int^ follicular (FO) B cells was similar between the two groups (T2: *p* = 0.03963, FO: *p* = 0.88202, and MZ: *p* = 0.03065) (Figure 5(a)). The cells with a CD19^+^CD5^+^CD1d^+^IL-10^+^ phenotype, that is, B10 cells, increased in SM-MSC-treated mice (*p* = 0.0277) (Figure 5(b)). Collectively, our results demonstrated that SM-MSCs induced increase in regulatory B subsets, comprising T2, MZ, and B10 cells, in the spleen of CIA mice.

## 4. Discussion

RA is a typically chronic and progressive autoimmune disease, which is associated with the breakdown of immune tolerance and with aberrant inflammatory responses. Although new therapeutic agents such as biologicals are now available, a considerable proportion of patients are still resistant to these therapies. MSC-based cell therapy is a promising option for patients with RA because of the anti-inflammatory, immunomodulatory, and regenerative properties of these cells. Appropriate sources of MSCs, ideal administration route, and optimal disease timing/stage are essential factors for achieving valid therapeutic effects.

In this study, human SM-MSCs were selected from among numerous cell sources because of their strong immunomodulatory properties during coculture with T lymphocytes in vitro and high proliferation capability with limited senescence [14]. Furthermore, the “off-the-shelf” property of MSCs makes the allogeneic human-derived MSCs available for murine CIA [26]. MSCs from the synovial membranes of osteoarthritic joints were found to be capable of suppressing CD4^+^ T cell proliferation upon CD3/CD28 stimulation, whereas BM-MSCs from the same patient could not [16]. In addition, human synovial membranes are an accessible source of MSCs. They are routinely removed in patients with OA, during arthroscopy and knee replacement surgery, which offers the advantage of excellent supply for future clinical applications. Moreover, previous research on SM-MSCs highlighted the important properties of these cells in tissue repair; these data suggest that SM-MSCs would be a promising option for the treatment of destructive diseases of the bone and cartilage [27].

To our knowledge, our study is the first to show the therapeutic effect of SM-MSCs in CIA. CIA is the most commonly studied murine model of RA and generally thought to be dependent on collagen-specific CD4^+^ T cells during the initial phase of autoimmune responses in the joints [28]. Repeated administration of SM-MSCs into inflamed joints could attenuate arthritis severity with reduction in inflammatory cytokines and increase in IL-10 production in serum, suggesting that local MSC treatment could exert a systemic therapeutic effect in mice. Recent studies reported that repeated intra-articular administration of allogeneic MSCs could be a safe strategy, leading to enhanced MSC availability [29]. Similarly, a study using proteoglycan-induced arthritis (PGIA), a well-studied inflammatory arthritis model, showed that intra-articular administration of BM-MSCs effectively reduced cumulative arthritis scores and PG-specific IgG2a antibody levels in the serum [30]. Another study using murine antigen-induced arthritis also addressed the systemic anti-inflammatory effect of MSCs, reflected in reduced TNF-*α* concentrations in serum following injection of BM-MSCs into the knee joints [31]. It has been well documented that MSCs exert immunoregulatory effects via locally cell-cell contact or secretion of soluble modulatory mediators, where in particular MSC-derived indoleamine 2,3-dioxygenase (IDO) in human and inducible nitric oxide synthase (iNOS) in mouse [32]. Besides, the modulatory effects of MSCs on immune responses, especially by means of secreting soluble factors, are critically linked to the “license” by inflammatory signals occurred in which MSCs are applied to [33]. We assumed that the interplay between exogenous MSCs and resident synoviocytes may trigger a series of biological processes participating in the paracrine actions in MSCs, which is most likely responsible for the therapeutic effect of MSCs in CIA.

Although our results are promising, a limitation of the current study is that we did not track the distribution of the injected SM-MSCs in vivo. Previous studies have shown that MSCs injected intra-articularly are retained at the injection site for 1–4 weeks, without migration to distant organs such as the lungs, spleen, and liver [30, 31].

Our data presented here suggest that the therapeutic effect of SM-MSCs in CIA mice was paralleled by increase in regulatory FoxP3^+^ T cells (Treg and Tfr) and induction of regulatory B cells (T2, MZ, and B10), both of which are essential for inhibiting dysregulated immune responses to self-antigens and are involved in self-tolerance mechanisms. Previous research documented that infusion of human gingiva-derived MSCs significantly ameliorated CIA via suppression of Th1 and Th17 responses and increase in FoxP3-expressing CD4^+^ T cells in the spleen [34], which is in agreement with our findings. In addition, SM-MSCs appeared to hamper the maturation and differentiation of B cells and induce the IL-10-competent regulatory B cells in our study. This was supported by the increase in immature-transitional stage B cells such as CD21^hi^CD23^hi^ transitional 2 (T2) cells and CD23^low^CD21^hi^ MZ cells, as well as CD5^+^CD1d^+^IL-10^+^ cells, in the spleens of SM-MSC-treated mice. Transfer of immature T2 cells from mice with arthritis at the remission stage could help recover the balance between Treg and Th1/Th17 responses in IL-10^−/−^ hosts [35, 36]. Similarly, adoptive transfer of CD5^+^CD1d^+^IL-10^+^ regulatory B cells prevented CIA development in mice with suppression of Th17 cells in the spleen and draining lymph nodes [25]. Clinical data also revealed that patients with new-onset RA had lesser IL-10-competent B cells, comprising CD19^+^CD5^+^CD1d^+^ cells and CD19^+^ TIM1^+^ cells, than healthy controls and that this decrease was positively correlated with the number of CD4^+^CD25^+^FoxP3^+^ Treg cells in peripheral blood [37]. Given the above and previous data, we hypothesize that, not only FoxP3^−^ Treg cells but also T2-, MZ-, and IL-10-expressing regulatory B cells were responsible for the suppression of inflammatory responses in mice with CIA, suggesting that the cellular interactions between regulatory B cells and Treg cells were pivotal in determining the beneficial outcome of SM-MSCs in CIA. A functional feed-forward loop between immunoregulatory T cells and B cells during SM-MSC treatment is of great interest and requires further study.

## 5. Conclusions

To our knowledge, the current study is the first to show that intra-articular injection of SM-MSCs could prevent arthritis development and suppress immune responses via expansion of FoxP3^+^ Treg cells and T2, MZ, and IL-10-competent regulatory B cells, thus recovering peripheral tolerance in mice with CIA. Our findings established the in vivo effect of SM-MSCs in CIA mice, indicating that intra-articular administration of SM-MSCs may constitute a potential approach for RA cell therapy.

## Figures and Tables

**Table 1:** Sequences for primers.

Target genes	Sequences (5′ to 3′)	
	Forward	Reverse
TNF-*α*	CAGGCGGTGCCTATGTCTC	CGATCACCCCGAAGTTCAGTAG
IFN-*γ*	TGAAAGACAATCAGGCCATC	TTGCTGTTGCTGAAGAAGGT
IL-17A	ATCCACCTCACACGAGGCACA	AGATGAAGCTCTCCCTGGACTC
IL-10	CCAGGGAGATCCTTTGATGA	CATTCCCAGAGG AATTGCAT
IL-4	GGTCTCAACCCCCAGCTAGT	GCCGATGATCTCTCTCAAGTGA
TGF-*β*	CTCCCGTGGCTTCTAGTGC	GCCTTAGTTTGGACAGGATCTG
*β*-actin	GGCTGTATTCCCCTCCATCG	CCAGTTGGTAACAATGCCATGT
